# Cost savings of outpatient versus inpatient hip and knee arthroplasty in Ontario, Canada

**DOI:** 10.1371/journal.pone.0320255

**Published:** 2025-05-08

**Authors:** Steven Habbous, Sisira Sarma, Brent Lanting, James Waddell, Erik Hellsten

**Affiliations:** 1 Ontario Health (Strategic Analytics), 525 University Ave., Toronto, Ontario, Canada; 2 Department of Schulich School of Medicine and Dentistry, Western University, London, Ontario, Canada; 3 ICES, Toronto, Ontario, Canada; 4 Division of Orthopedic Surgery, St. Michael’s Hospital, Toronto, Ontario, Canada; Taipei Medical University, TAIWAN

## Abstract

**Introduction:**

Hip and knee replacement surgery is one of the most commonly performed elective procedures, accounting for significant healthcare costs and resource utilization. In recent years, the proportion of hip and knee arthroplasties performed in outpatient settings has grown rapidly. Although the safety and effectiveness of outpatient vs. inpatient hip and knee arthroplasty have been documented in the literature, estimates of health system cost-savings in Canada are limited.

**Methods:**

We employed a population-based retrospective cohort study design. We obtained data on patients aged 18–105 years who underwent hip or knee replacement surgery in both inpatient and outpatient settings in Ontario, Canada between 2018/19 and 2022/23. Patients who underwent outpatient hip and knee arthroplasty were matched to inpatient cases using a propensity score based on age, sex, comorbidity, area-level sociodemographic factors, total/partial replacement, and surgery date. We analyzed cost data that included hospitalization and ambulatory care visits, physician billing, home care, and oral medications. We utilized generalized linear models to identify the best fit regression model and estimated the average cost-savings associated with outpatient versus inpatient arthroplasty during the preoperative (30-days before surgery), perioperative (surgery + 30 days), 1–6-month postoperative, 6–12-month postoperative, 12–24-month postoperative, and 24–36-month postoperative periods. The costs were reported in 2023 Canadian dollars.

**Results:**

A total of 35,894 hip arthroplasty patients and 49,597 knee arthroplasty patients were included in our analysis. During the perioperative period, outpatient arthroplasty was less costly than inpatient arthroplasty for hip replacement by $3,859 (95% CI -$4045, -$3745) and for knee replacement by $3,966 ($-4080, $-3851). Over 3 years of follow-up, outpatient arthroplasty was less costly than inpatient arthroplasty for hip replacement by $7058 (-$8086, -$6031) and for knee replacement by $7043 (-$7842, -$6243).

**Conclusion:**

Outpatient hip and knee arthroplasty is cost-saving both during and beyond the perioperative period in comparison with similar patients who undergo inpatient arthroplasty in Ontario. Policies should be put in place to incentivize continued uptake of outpatient arthroplasty, which we estimate could save the Ontario healthcare system up to $98 million per year.

## Introduction

Hip and knee replacement surgery is one of the most commonly performed elective procedures in Canada, accounting for significant healthcare spending and resource utilization. In Ontario, Canada’s most populous province, pressures on hospitals associated with the COVID-19 pandemic resulted in a dramatic increase in the proportion of hip and knee arthroplasties performed in the outpatient setting, which may have had positive impacts on patients and the broader healthcare system [1].

Several studies have demonstrated equivalent clinical outcomes between the two settings. One matched study from Ontario revealed that there was no difference in the risk of revision, post-operative infection, or death within 1 year of surgery (<2% of patients experienced any event in either group) [2]. Other studies have demonstrated comparable safety and effectiveness profiles between outpatient and inpatient hip/knee arthroplasty in the United States [3].

Outpatients were also found to have similar quality-of-life estimates compared to inpatients. One randomized controlled trial reported similar pain and stiffness at 12 weeks and a better (higher) functional score [4]. Similar findings were reported in a small (n=84) randomized controlled trial from China, but the effects on quality of life depended on the instrument used [5]. Given the similar clinical and quality-of-life outcomes between patients selected for outpatient and inpatient hip/knee replacement, a costing analysis would be informative for health system planning and healthcare resource allocation decisions.

### Literature Review

A recent review from the Canadian Agency for Drugs and Technologies in Health (CADTH) concluded that there exists a considerable gap in the literature concerning the total cost-savings between inpatient and outpatient hip and knee replacement, particularly for total knee arthroplasty (TKA) [6]. A model-based US economic evaluation study found that inpatient total hip arthroplasty (THA) was not cost-effective compared with outpatient THA [7]. Using individual-patient data, one small (n=115) randomized controlled trial from a single centre in London, Ontario, demonstrated that outpatient THA was cost-saving in the as-treated population for direct health system costs [mean difference −1329.53 (95% CI −2473.39 to −185.67) in 2019 Canadian dollars] and from the societal perspective when unpaid time off and caregiver assistance costs were included [mean difference −2578.80 (95% CI −5071.08 to −86.51)] [4]. One small observational study (n=50) from a single centre and a single surgeon in Montreal, Canada reported lower costs for outpatient THA for select resource use between pre-operative assessment and 6 weeks post-operatively [mean difference $1239] [8]. Another small (n=40) single-centre single-surgeon study reported mean cost savings of $3156 with no difference in clinical outcomes [9]. One population-based cost analyses in the literature reported cost savings of $1,721 per total joint replacement, but the study data consisted of an older population (January 2019 to March 2021), used aggregated data, and had limited cost data [10].

The purpose of the present study was to fill the knowledge gap on the cost savings associated with outpatient versus inpatient hip and knee arthroplasty.

## Methods

### Setting

In Canada, all hospital and physician services deemed medically necessary are publicly funded by provincial governments. All hip/knee replacement surgeries are performed in hospitals and data are mandated to be collected by the Canadian Institute of Health Information (CIHI).

### Patient cohorts

**Fig 1:**
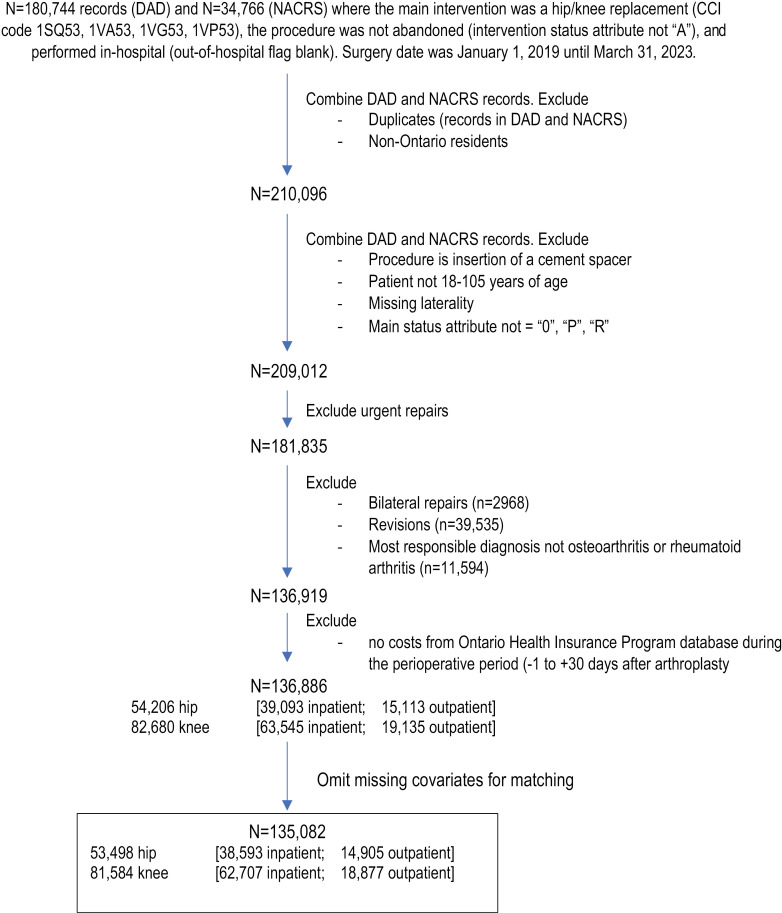
Cohort creation. Patients were identified from the Discharge Abstract Database (DAD; hospitalizations) and the National Ambulatory Care Reporting System (NACRS; ambulatory hospital visits).

Inpatient and outpatient hip/knee arthroplasty data were obtained from the hospital Discharge Abstract Database (DAD) and the National Ambulatory Care Reporting System (NACRS) managed by the Canadian Institute of Health Information (CIHI), respectively, as previously described (Fig 1) [2]. We included Ontario residents aged 18–105 years who received an elective (e.g., non-urgent) hip or knee replacement between April 1, 2019 and March 31 2023. Only primary arthroplasties with a most responsible diagnosis related to either osteoarthritis or rheumatoid arthritis were included. Additionally, we excluded patients who had no physician billing records (or a cost of $0) in the Ontario Health Insurance Program (OHIP) database during the perioperative period (the day before surgery until day 30 after surgery, inclusive). This was done because hip/knee arthroplasty is an OHIP-funded procedure for all Ontarians, so the lack of preceding or subsequent records suggests that those patients may be non-Ontario residents.

Patients were categorized as outpatients if the procedure was captured in NACRS or if the patient was discharged on the same day of the procedure (captured in DAD where the discharge date equals the admission date). Inpatient procedures were captured in the DAD if not a same-day discharge.

### Covariates

Comorbidities were estimated from inpatient and outpatient hospital records using ICD-10 diagnostic codes with a look-back period of 3 years before the surgery date. Diabetes was supplemented using the Ontario Drug Benefits (ODB) with evidence of any oral medication related to diabetes management. The ODB captures oral medications for Ontarians aged 65+ or who are receiving social assistance.

Census dissemination area-level marginalization indices were obtained from the Ontario Marginalization Index using the patients’ postal code at the time of surgery (from DAD or NACRS). The Ontario Marginalization Index combines dissemination area-level sociodemographic characteristics from the 2016 Census, and we considered all four domains of area-level marginalization: 1) material resources (formerly material deprivation); 2) households and dwellings (formerly residential instability); 3) the age and labor force (formerly dependency); and 4) racialized/newcomer (formerly ethnic diversity).

### Costs

Costs were captured from all available databases and reported in 2023 Canadian dollars, converted using the Consumer Price Index as recommended by CADTH [11]. We estimated the total costs for any healthcare encounter incurred by patients in six distinct periods (Table 1). Costs were obtained from inpatient (CIHI-DAD) and outpatient (CIHI-NACRS) records using the Ontario Case Costing Initiative, the physician billing database (OHIP), the prescription medication costs for seniors and social assistance recipients (ODB), rehabilitation care data (National Rehabilitation Reporting System; NRS), and the home care database by multiplying the number of visits or hours by their corresponding unit costs derived from the Ontario Healthcare Financial and Statistical System. Detailed definitions can be found in the Technical Appendix (S1 Appendix).

**Table 1:** Definition of time periods.

Time Period	Description	Cut-off date for arthroplasty^a^
Preoperative	30 days before surgery until the day before surgery (not including this date)	March 31 2023
Perioperative	the day before surgery until day 30 after surgery (inclusive)	March 31 2023
1-6 months post-operative	day 31 to day 183	March 31 2023
6-12 months post-operative	day 184 to day 365	September 30, 2022
Year 2 post-operative	day 365 to day 730	September 30, 2021
Year 3 post-operative	day 731 to day 1095	September 30, 2020

^a^ September 30, 2023 was the most recent date where all costing data were available. Thus, this was treated as the censor date and only patient receiving an arthroplasty between April 1, 2019 and the indicated cut-off date was used for analyzing costs in the specified period

### Statistical methods

To account for potential selection bias, outpatients were matched to inpatients using inverse probability of treatment weight (IPTW) derived from a logistic regression (conducted separately for knee and hip replacement) on age, sex, Charlson comorbidity score, rurality, Ontario marginalization index quintiles, total/partial replacement, and date of surgery [12]. A caliper of 0.05 was chosen and balance was assessed using standardized mean differences using the MatchIt() package in R. Balance was considered met when the absolute value of the standardized mean difference was <0.1 (S1 Table in S1 File). After excluding patients with non-overlapping IPTWs, 95% of outpatient hip patients and 99% of outpatient knee patients were included in the IPTW cohort.

Regression models were fit using the IPTW cohort in STATA using the code provided by Jones et al. [13], adjusted for age centered at the mean, sex, comorbidity score, rurality, marginalization index quintiles, total/partial replacement, and fiscal year of surgery. All the models used weights produced by the IPTW estimator and cluster-robust standard errors that account for the within-cluster dependence of observations (due to matching up to 2 inpatients per 1 outpatient) to generate heteroskedasticity-consistent standard errors (SEs) for inference. For modeling costs, we used generalized linear models with a range of link functions and distributional families, including linear (link=identity/family=Gaussian), square-root/gamma, log/gamma, log/normal, and log/Poisson. Note that less than 7% of patients in our sample incurred zero costs in any time period, so two-part cost models were not needed [14]. As a robustness check, we also considered the inverse-probability-weighted regression adjustment (IPW-RA) methodology using Poisson regression for modelling the outcome and logistic regression for modeling the IPW [15–17]. To choose the best-fitting model, we considered ‘badness-of-fit’ tests (a higher p-value is better), including Pregibon’s link-test, Pearson’s p-value, and the modified Hosmer-Lemeshow test for model specification [13]. We used the Copas test to assess overfitting following k-fold cross-validation (k=100). We also compared ‘goodness-of-fit’ estimates, including the root-mean square error (RMSE; lower is better), mean absolute prediction error (MAPE; smaller is better), mean prediction error (MPE) for bias (0 is better), and R^2^ (closer to 1 is better). For each outcome, the best-performing model was chosen and the average marginal effect (AME) of each variable in the model was reported on the raw (dollar) scale, which represents the effect of a 1-unit change in the predictor on the outcome. Throughout, we used a p-value cut-point of 0.05 for determining statistical significance. The AME and SE for each time period were combined to calculate the total expected effects of outpatients versus inpatients arthroplasty on costs over the 3-year time period as $ME_{total}=\sum ME$ and $SE_{total}=\sqrt{\sum SE^{2}}$.

### Software and privacy

Research ethics was not required as per the Ontario Health privacy assessment as this work was performed for the purpose of quality improvement and no identifying information was obtained. This study was compliant with section 45(1) of PHIPA (Ontario Health is a prescribed entity); thus, patient consent was not required.

Data were extracted in March 2024 using Statistical Analysis Software (SAS v9.4), propensity score weighting methods were performed using R v4.2.1, and model-fitting was performed using STATA v18.0.

## Results

In the IPTW cohort, 35,894 hip arthroplasty patients and 49,597 knee arthroplasty patients were included. At the time of surgery, the hip arthroplasty patients had a mean age of 66 years, 50% were male, and 87% had no comorbidities (S1 Table in S1 File). Knee arthroplasty patients had a mean age of 67 years at the time of surgery, 42% were male, and 80% had no comorbidities (S1 Table in S1 File).

### Hip Arthroplasty Costs

**Fig 2:**
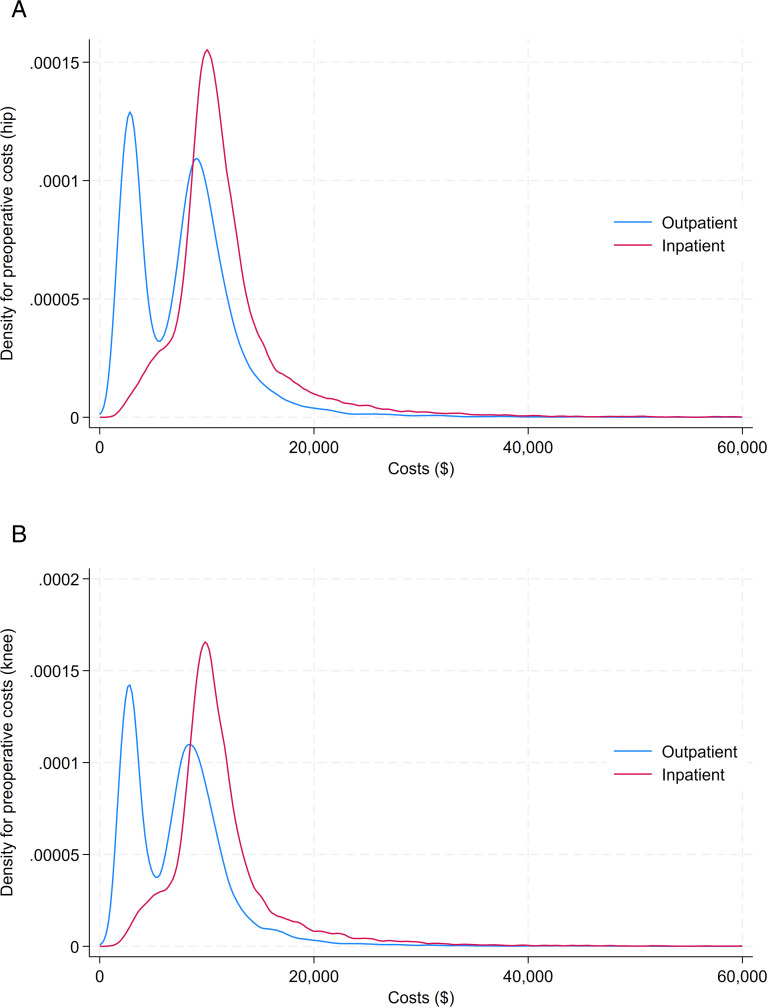
Distribution of perioperative costs by setting. Total costs for inpatient and outpatient patients receiving (A) hip arthroplasty; and (B) knee arthroplasty.

The mean cost for hip arthroplasty during the perioperative period was $12,057 ($8,539) for inpatients and $7,958 ($5,650) for outpatients (Table 2). The distribution of perioperative costs was bimodal for outpatients only (Fig 2a). After adjustment, outpatient arthroplasty was significantly less costly than inpatient arthroplasty by $3,894 (95% CI -$4,045, -$3,745), p<0.0001. Other factors associated with higher costs included older age ($139 per 10 years), higher comorbidity score ($2,799 for 3+ versus 0), patient residence in areas having more racialized people or newcomers ($1,097 for most versus least) and patient residence in areas with greater marginalization related to age and labor force ($379 for highest versus lowest) (Table 3).

**Table 2:** Costs by joint type, setting, and time period.

	Hip arthroplasty			Knee arthroplasty		
	Inpatient	Outpatient	AME (SE), $^b^	Inpatient	Outpatient	AME (SE), $^b^
**Preoperative (-30 to -2 days)**						
N	21742	14093	**-107.45**	30828	18769	**-91.61**
Mean (SD)	$505 ($1107)	$376 ($926)	**(10.58)**	$528 ($1099)	$418 ($1198)	**(8.65)**
Median (Q1, Q3)	$291 [$162, $487]	$204 [$926, $372]	**p<0.0001**	$320 [$171, $526]	$242 [$1198, $432]	**<0.0001**
**Perioperative (-1 to** +**30 days)**						
N	21742	14093	**-3894.94**	30828	18769	**-3965.69**
Mean (SD)	$12057 ($8539)	$7958 ($5650)	**(76.44)**	$11571 ($6769)	$7523 ($5495)	**(58.57)**
Median (Q1, Q3)	$10692 [$9045, $13063]	$8094 [$5650, $10365]	**p<0.0001**	$10351 [$8791, $12522]	$7431 [$5495, $9749]	**<0.0001**
**1 to 6 months postoperative**						
N	21742	7764	**-551.82**	30828	18769	**-469.91**
Mean (SD)	$2624 ($5992)	$1933 ($5040)	**(55.58)**	$2846 ($5993)	$2236 ($4748)	**(47.36)**
Median (Q1, Q3)	$1003 [$468, $2249]	$781 [$5040, $1675]	**p<0.0001**	$1193 [$589, $2612]	$1017 [$4748, $2125]	**<0.0001**
**6 to 12 months postoperative**						
N	12686	5318	**-439.13**	15045	8791	**-408.88**
Mean (SD)	$2141 ($5910)	$1640 ($5183)	**(138.13)**	$2430 ($8647)	$2013 ($5384)	**(74.36)**
Median (Q1, Q3)	$523 [$140, $1609]	$372 [$5183, $1174]	**p<0.0001**	$674 [$209, $1860]	$595 [$5384, $1669]	**<0.0001**
**12 to 24 months postoperative**						
N	9265	1623	**-1096.62**	9969	5543	**-983.05**
Mean (SD)	$4511 ($10857)	$3443 ($8514)	**(247.98)**	$5309 ($11717)	$4571 ($10755)	**(180.61)**
Median (Q1, Q3)	$1300 [$413, $3697]	$1050 [$8514, $2733]	**p<0.0001**	$1692 [$597, $4463]	$1523 [$10755, $3850]	**<0.0001**
**24 to 36 months postoperative**						
N with costs (complete case)	3810	0	**-968.57**	2946	1031	**-1123.80**
Mean (SD)	$4251 ($9819)	$3528 ($10443)	**(430.33)**	$5060 ($11949)	$4383 ($10537)	**(350.21)**
Median (Q1, Q3)	$1191 [$374, $3414]	$939 [$10443, $2665]	**p=0.01**	$1539 [$518, $3951]	$1540 [$10537, $3724]	**0.001**

^a^ restricted to time period where complete data are expected

^b^ marginal effect of outpatient versus inpatient setting, adjusted for centered age, sex, comorbidity score, rurality, total/partial replacement, and area-level deprivation, dependency, instability, and ethnic concentration quintiles.

**Table 3:** Regression of costs during the perioperative period.

	Hip arthroplasty			Knee arthroplasty		
	**AME** ^a^	**95% CI**	**p-value**	**AME** ^a^	**95% CI**	**p-value**
Age (per 10 years)^b^	$139	($64, $214)	<.0001	$150	($78, $221)	<.0001
**Outpatient vs inpatient**	$**-3895**	($**-4045,** $**-3745)**	**<.0001**	$**-3966**	($**-4080,** $**-3851)**	**<.0001**
Male vs female	$-94	($-242, $54)	0.21	$-121	($-233, $-8)	0.04
Urban vs rural	$238	($-21, $497)	0.07	$-22	($-199, $155)	0.81
Material resources (vs least)						
2	$67	($-136, $270)	0.52	$152	($-7, $311)	0.06
3	$235	($-7, $476)	0.06	$226	($53, $398)	0.01
4	$321	($25, $616)	0.03	$177	($-4, $358)	0.06
5 (most marginalized)	$256	($-6, $519)	0.06	$355	($156, $554)	<.0001
Racialized/newcomer (vs least)						
2	$-102	($-319, $114)	0.36	$189	($5, $372)	0.04
3	$327	($54, $600)	0.02	$311	($120, $501)	0.001
4	$430	($166, $694)	0.001	$571	($370, $773)	<.0001
5 (highest density)	$1097	($807, $1388)	<.0001	$551	($350, $752)	<.0001
Age/labor force (vs least)						
2	$94	($-118, $307)	0.38	$138	($-46, $322)	0.14
3	$194	($-45, $432)	0.11	$96	($-102, $295)	0.34
4	$280	($52, $508)	0.02	$-21	($-211, $169)	0.83
5 (most marginalized)	$379	($113, $644)	0.005	$134	($-66, $335)	0.19
Households and dwellings (vs least)						
2	$-196	($-399, $7)	0.06	$121	($-55, $298)	0.18
3	$38	($-206, $282)	0.76	$53	($-115, $221)	0.54
4	$-76	($-317, $166)	0.54	$86	($-103, $275)	0.37
5 (most marginalized)	$223	($-30, $476)	0.08	$343	($138, $548)	0.001
Total vs partial replacement	$-453	($-1356, $451)	0.33	$485	($-8, $977)	0.05
Comorbidity score (vs 0)						
1	$688	($473, $904)	<.0001	$473	($336, $610)	<.0001
2	$1718	($1080, $2356)	<.0001	$1244	($910, $1579)	<.0001
3+	$2799	($1566, $4032)	<.0001	$2087	($1350, $2824)	<.0001
Fiscal year (vs 2019)						
2020	$-388	($-628, $-147)	0.002	$394	($155, $634)	<.0001
2021	$-645	($-904, $-386)	<.0001	$154	($-86, $395)	0.20
2022	$-18	($-187, $151)	0.84	$295	($101, $489)	<.0001

^a^ average marginal effect (AME) with 95% confidence interval (CI) following a log-normal generalized linear model

^b^ age was centered by subtracting the mean

Compared with inpatients, outpatients incurred fewer costs during the preoperative period [-$107 (SE 10.6)], 1–6-month postoperative period [-$552 (SE 55.6)], 6–12-month postoperative period [-$439 (SE 138)], 12–24-months postoperative period [-$1097 (SE 248)], and the 24–36-month postoperative period [-$969 (SE 430)] (S2 Table in S1 File).

Summing over all time periods, the average cost difference between outpatients and inpatients was -$7,058 [-$8,086, -$6,031] over three years.

### Knee Arthroplasty Costs

The mean cost of knee arthroplasty during the perioperative period was $11,571 (SD $6,769) for inpatients and $7,523 ($5,495) for outpatients (Table 2). The distribution of perioperative costs was bimodal for outpatients only (Fig 2b). After adjustment, outpatient arthroplasty was significantly less costly than inpatient arthroplasty by $3,966 ($-4,080, $-3,851), p<0.0001. Other factors associated with higher costs included older age ($150 per 10 years), higher comorbidity ($2,087 for 3+ versus 0), residing in more ethnically diverse areas ($551 for most versus least diverse), areas of lower material resources ($355 for most vs least marginalized), and greater residential marginalization ($343 for most versus least marginalized)

Compared with inpatients, outpatients incurred fewer costs during the preoperative period [-$91 (SE 8.6)], 1–6-month postoperative period [-$470 (SE 47.4)], 6–12-month postoperative period [-$409 (SE 74.4)], 12–24-month postoperative period [-$983 (SE 181)], and 24–36-month postoperative period [-$1,124 (SE 350)] (S3 Table in S1 File).

Summing over all time periods, the average cost difference between outpatients and inpatients was -$7,043 [-$7,842, -$6,243] over three years.

### Sensitivity analysis

The costs of all patients before matching are reported in Supplementary Table S4 in S1 File, demonstrating that the propensity-score matching excluded more high-cost inpatients (e.g., complex patients likely ineligible for outpatient arthroplasty). Using the IPW-RA models, all the models demonstrated that outpatient hip/knee arthroplasty was cost-saving, but most treatment effects were more cost-saving than the models reported above (S5 Table in S1 File).

## Discussion

In this study, we found that outpatient hip and knee arthroplasty is less costly than inpatient procedures in appropriately selected patients. During the perioperative period alone, outpatient arthroplasty yielded a savings of $3,859 (hip) and $3,966 (knee) for the average patient in comparison with inpatient arthroplasty.

In the 2022/23 fiscal year, a total of 9,056 inpatient hip and 15,783 inpatient knee replacements were performed in the IPTW-matched cohort (e.g., potentially eligible for outpatient arthroplasty). Thus, a total of $97.5 million could have been saved if all these inpatient procedures were eligible and performed in the outpatient setting instead (9,056×$3,859 + 15,783×$3,966). Although these represent upper limits, even if we were to assume that half of those potentially eligible inpatient procedures could have been performed in the outpatient setting, this still amounts to $48.8 million in total cost savings for the 2022/23 fiscal year alone.

With the aging of the Ontario population, the demand for hip/knee arthroplasty is expected to increase; hence, the potential for health system cost-savings from the adoption of outpatient arthroplasty procedures is expected to increase. During the perioperative period alone, every two inpatient procedures converted to the outpatient setting (net savings $8,000) theoretically yielded cost savings sufficient to pay for one additional outpatient procedure (mean cost $8,000). By eliminating the need for a hospital bed, the availability of beds ceases to be a limiting factor. If a higher rate of outpatient arthroplasty procedures can be performed over time, we may also expect to see a concomitant reduction in the time until surgery for some patients. Reduced wait times may have economic benefits for patients, who tend to incur the most out-of-pocket expenses during this time (e.g., caregivers at home and lost wages) [18,19]. In addition, patients may prefer to recover at home rather than at the hospital. An additional benefit is the potential to reallocate the saved dollars of converting inpatient procedures to outpatient procedures. Hospital funding incentives should therefore favour outpatient procedures while maintaining quality standards.[20]. Thus, without compromising patient outcomes, shifting more hip and knee arthroplasties to outpatient settings appears to be favourable from both a cost and patient experience standpoint.

### Barriers to outpatient care

Despite these potential benefits, some barriers to outpatient arthroplasty still must be overcome. One challenge is enabling access to same-day analgesia and the design and maintenance of an outpatient clinic [2,20]. For example, access to after-hours physiotherapy can be used to help outpatients meet the necessary discharge criteria and make it home after their procedure [21,22]. Where after-hours physiotherapy is challenging, telerehabilitation strategies may be effective, as demonstrated by one small (n=142) Spanish study following knee replacement [23]. There is also a need for consistent messaging and education, in addition to effective means to check-in with patients after they have left the hospital. One study in the United Kingdom demonstrated positive patient and provider satisfaction in addition to resource savings due to standardized virtual care [24]. Lastly, there is a growing number of patients needing hip or knee arthroplasty who have more comorbidities. As programs and surgeons increasingly adopt outpatient surgery, they may increasingly offer outpatient treatment to more patients with some comorbidity, so active monitoring of patient outcomes over time is critical to ensure continued success with the outpatient model. Regional variation is also important to consider, including 1) variability in access to regional anesthetic techniques and drug administration; and 2) some programs refer patients to a centralized intake which subsequently distributes patients to providers with the shortest wait list rather than geographic approximation, which may be a barrier for patients who prefer not to travel to receive treatment [25]. Despite favourable outcomes for patients and hospitals (e.g., low return to the emergency department or re-admissions), the financial gain is felt primarily by hospitals. Surgeons themselves may not benefit since there is no change in billing by setting, no post-operative rounding making post-operative follow-up challenging, and no change in surgical volume. Anecdotally, there is an assumption of greater risk for outpatient procedures (e.g., the surgeon is responsible for a return to the emergency department; providing virtual care immediately post-operatively is challenging; and greater care is needed on the day of surgery). Thus, transition to outpatient care may be perceived to have created some conflict, where the hospital is gaining from the initiative, but is unwilling to increase volume or re-invest in care with the saved resources (e.g., refurbishing the existing clinic). Instead, hospitals reroute those saved dollars to programs working at a deficit, which may not align with the best interests of the orthopedics department and therefore disincentivize outpatient programs.

### Anecdotal experience

With substantial hospital-level variation in the adoption of outpatient hip/knee replacements [2], why did some hospitals transition to outpatient care? Some hospitals began the transition early (well before the pandemic) because of pressures driven by the limited availability of hospital beds. Others had a different experience: forced by the COVID-19 pandemic to either perform outpatient procedures or not at all, some hospitals quickly adopted outpatient arthroplasty. Sharing the experience from one program, the hospital was enthusiastic and fully supported the program; surgeons were happy to have the work; and patients understood the option to either go home the same day or wait >6 months until a bed became available. The program is happy with the current paradigm, performing >50% of all procedures on an outpatient basis with positive patient satisfaction. With a protocol in place with access to physiotherapy, nursing, and anesthesiology, this paradigm is now a permanent fixture of the hospital and is unlikely to regress to pre-pandemic levels.

## Limitations

Despite matching on several sociodemographic and clinical characteristics, we cannot completely eliminate confounding factors by indication where healthier patients and those with fewer comorbidities are differentially favoured to receive outpatient arthroplasty. We also could not account for cases that were originally planned for outpatient arthroplasty but ended up switching to inpatient arthroplasty when the need arose. From an intention to treat perspective, the inpatient cohort would include all the intended outpatient cases that admitted with resultant higher costs. However, in addition to propensity-score matching, our regression models also included all available covariates in the models, and we do not expect our conclusions to change despite these limitations. A second limitation is the unavailability of other costs, including those associated with long-term care, home and caregiver costs, and lost wages. Other missed costs include the costs associated with setting up an outpatient clinic for hip and knee arthroplasty procedures. Another limitation is the potential for misspecification or overfitting for some of the regression models used. In particular, it was difficult to find a good-fitting model for the perioperative period, which may be due to the bimodal nature of the cost distribution. Nevertheless, we do not believe these issues will have a substantial impact on the conclusions of this study, particularly with our large sample size which may be robust to some violations of model assumptions.

Finally, although follow-up beyond the perioperative period increased the total cost savings, this was unexpected since pre-specified outcomes in the literature were similar between groups [2]. Rather than attributing this to the superior performance of outpatient arthroplasty, this may instead be driven by residual confounding not accounted for by the variables available for matching and adjustment. However, in appropriately selected patients, outpatient arthroplasty is cost-saving from the perspective of the healthcare system and we expect these findings to be generalizable to jurisdictions with healthcare systems similar to Ontario.

## Conclusion

In this population-based retrospective cohort study, we fill an important knowledge gap on the cost-savings associated with outpatient versus inpatient hip and knee arthroplasty procedures. The potential cost savings to the health care system are estimated to be $3,859 per hip arthroplasty and $3,966 per knee arthroplasty, resulting in up to $98 million per year total cost savings. Policies should encourage outpatient hip and knee arthroplasty models of care by incentivizing hospitals and surgeons, with some of these cost savings recuperated by the orthopedics programs. Such a redesign may be expected to translate to more patients receiving timely arthroplasty care.

## Supporting information

S1 File Supplementary Tables for balance after matching (Table S1) ; full regression marginal effects for hip (Table S2) and knee (Table S3); costs in the unmatched cohort (Table S4); and model sensitivity analysis (Table S5).
(DOCX)

S1 Appendix Technical Appendix descriptive the data sources used for cost analysis. (DOCX)

S1 Checklist STROBE checklist for observational cohort studies. (DOCX)
