# Supplementary material for: Cost savings of outpatient versus inpatient hip and knee arthroplasty in Ontario, Canada
Source: PLoS One. 2025 May 8;20(5):e0320255. doi: 10.1371/journal.pone.0320255 (PMC12061100; doi:10.1371/journal.pone.0320255)
Supplement: S1 File — ; full regression marginal effects for hip (Table S2) and knee (Table S3); costs in the unmatched cohort (Table S4); and model sensitivity analysis (Table S5). (DOCX) [file pone.0320255.s001.docx]

# Supporting information

**Table S1. Balance after matching for cost analysis.** Descriptive statistics are shown between groups. An absolute standardized mean difference < 0.1 indicates balance.

**Table S2: Full regression marginal effects for HIP arthroplasty by time period.** The average marginal effect (standard error) is shown for the effect of each covariate on the total costs incurred during the specified time period.

**Table S3: Full regression marginal effects for KNEE arthroplasty by time period.** The average marginal effect (standard error) is shown for the effect of each covariate on the total costs incurred during the specified time period.

**Table S4: Costs in the unmatched cohort by period and joint type**.

**Table S5: Model sensitivity analysis.**

# Supplementary Table S1: Balance after matching for costing analysis

|  | **Hip cohort** | | | **Knee cohort** | | |
| --- | --- | --- | --- | --- | --- | --- |
|  | **Inpatient (N=21801)** | **Outpatient (N=14093)** | **SMD^a^** | **Inpatient (N=30958)** | **Outpatient (N=18769)** | **SMD^a^** |
| Age, years) | 67.0 (10.9) | 65.5 (9.79) | -0.0375 | 68.2 (8.90) | 67.0 (8.35) | -0.055 |
|  |  |  |  |  |  |  |
| Sex |  |  |  |  |  |  |
| Female | 11407 (52.3%) | 7044 (50.0%) | -0.0145 | 18184 (58.7%) | 10698 (57.0%) | -0.0129 |
| Male | 10394 (47.7%) | 7049 (50.0%) | 0.0145 | 12774 (41.3%) | 8071 (43.0%) | 0.0129 |
|  |  |  |  |  |  |  |
| Rurality |  |  |  |  |  |  |
| Rural | 3602 (16.5%) | 2362 (16.8%) | -0.0031 | 4744 (15.3%) | 2858 (15.2%) | -0.0023 |
| Urban | 18199 (83.5%) | 11731 (83.2%) | 0.0031 | 26214 (84.7%) | 15911 (84.8%) | 0.0023 |
|  |  |  |  |  |  |  |
| Material resources (formerly material deprivation) | | | | | | |
| 1 (most resources) | 6180 (28.3%) | 4265 (30.3%) | 0.0163 | 7201 (23.3%) | 4433 (23.6%) | 0.0046 |
| 2 | 4973 (22.8%) | 3283 (23.3%) | 0.003 | 6778 (21.9%) | 4127 (22.0%) | -0.0007 |
| 3 | 4316 (19.8%) | 2700 (19.2%) | -0.0104 | 6402 (20.7%) | 3920 (20.9%) | -0.0008 |
| 4 | 3572 (16.4%) | 2224 (15.8%) | -0.0012 | 5836 (18.9%) | 3489 (18.6%) | -0.0017 |
| 5 (fewest resources) | 2760 (12.7%) | 1621 (11.5%) | -0.0136 | 4741 (15.3%) | 2800 (14.9%) | -0.0019 |
|  |  |  |  |  |  |  |
| Households and dwellings (formerly residential instability) | | | | | | |
| 1 (most resources) | 3826 (17.5%) | 2647 (18.8%) | 0.0174 | 6101 (19.7%) | 3953 (21.1%) | 0.0111 |
| 2 | 4923 (22.6%) | 3315 (23.5%) | 0.0089 | 6873 (22.2%) | 4293 (22.9%) | 0.0086 |
| 3 | 4849 (22.2%) | 3134 (22.2%) | -0.0037 | 6904 (22.3%) | 4128 (22.0%) | -0.0041 |
| 4 | 4127 (18.9%) | 2577 (18.3%) | -0.0124 | 5772 (18.6%) | 3399 (18.1%) | -0.004 |
| 5 (fewest resources) | 4076 (18.7%) | 2420 (17.2%) | -0.0117 | 5308 (17.1%) | 2996 (16.0%) | -0.0134 |
|  |  |  |  |  |  |  |
| Age and labor force (formerly dependency) | | | | | | |
| 1 (least marginalized) | 3461 (15.9%) | 2423 (17.2%) | 0.0151 | 5549 (17.9%) | 3571 (19.0%) | 0.0075 |
| 2 | 3805 (17.5%) | 2512 (17.8%) | 0.0019 | 5470 (17.7%) | 3435 (18.3%) | 0.0078 |
| 3 | 4219 (19.4%) | 2779 (19.7%) | 0.0013 | 5752 (18.6%) | 3536 (18.8%) | 0.0033 |
| 4 | 4250 (19.5%) | 2648 (18.8%) | -0.0104 | 5913 (19.1%) | 3474 (18.5%) | -0.0045 |
| 5 (most marginalized) | 6066 (27.8%) | 3731 (26.5%) | -0.0068 | 8274 (26.7%) | 4753 (25.3%) | -0.0127 |
|  |  |  |  |  |  |  |
| Racialized/newcomer (formerly ethnic diversity) | | | | | | |
| 1 (lowest density) | 5882 (27.0%) | 3763 (26.7%) | -0.0041 | 7882 (25.5%) | 4630 (24.7%) | -0.0059 |
| 2 | 5001 (22.9%) | 3231 (22.9%) | 0.0027 | 6542 (21.1%) | 4008 (21.4%) | 0.0096 |
| 3 | 4561 (20.9%) | 2995 (21.3%) | 0.005 | 5840 (18.9%) | 3505 (18.7%) | -0.0021 |
| 4 | 3917 (18.0%) | 2558 (18.2%) | 0.0017 | 5121 (16.5%) | 3025 (16.1%) | -0.0086 |
| 5 (highest density) | 2440 (11.2%) | 1546 (11.0%) | -0.0066 | 5573 (18.0%) | 3601 (19.2%) | 0.0064 |
|  |  |  |  |  |  |  |
| Repair type |  |  |  |  |  |  |
| Partial | 86 (0.4%) | 106 (0.8%) | 0.0213 | 316 (1.0%) | 276 (1.5%) | 0.0212 |
| Total | 21715 (99.6%) | 13987 (99.2%) | -0.0213 | 30642 (99.0%) | 18493 (98.5%) | -0.0212 |
|  |  |  |  |  |  |  |
| Charlson comorbidity score | | | | | | |
| 0 | 18385 (84.3%) | 12269 (87.1%) | 0.0125 | 23897 (77.2%) | 15224 (81.1%) | 0.0438 |
| 1 | 2922 (13.4%) | 1543 (10.9%) | -0.019 | 5911 (19.1%) | 2998 (16.0%) | -0.0412 |
| 2 | 384 (1.8%) | 221 (1.6%) | 0.0117 | 930 (3.0%) | 439 (2.3%) | -0.0125 |
| 3+ | 110 (0.5%) | 60 (0.4%) | 0.0039 | 220 (0.7%) | 108 (0.6%) | -0.0021 |
|  |  |  |  |  |  |  |
| Surgery date^b^ | 18963.6 | 18969.96 | -0.0181 | 19047.27 | 19057.75 | -0.0345 |
| Distance (matching) | 0.3745 | 0.3709 | 0.0211 | 0.3471 | 0.3447 | 0.0153 |
|  |  |  |  |  |  |  |
| Matching process |  |  |  |  |  |  |
| All | 38593 | 14905 |  | 62707 | 18877 |  |
| Matched (ESS) | 19397.66 | 14093 |  | 27794.02 | 18769 |  |
| Matched | 21801 | 14093 |  | 30958 | 18769 |  |
| Unmatched | 16792 | 812 |  | 31749 | 108 |  |
| Discarded | 0 | 0 |  | 0 | 0 |  |
|  |  |  |  |  |  |  |
| % matched |  | 14093/14905 (95%) | |  | 18769/18877 (99%) | |
| ^a^ SMD (standardized mean difference) accounts for weights produced in the matching process. Significant difference in SMD was considered if \|SMD\| > 0.1  ^b^ date stored as a number  ESS – effective sample size | | | | | | |

# Supplementary Table S2: Full regression marginal effects for hip arthroplasty

|  | Preoperative period | | 1-6 months postoperative period | | 6-12 months postoperative period | | 12-24 months postoperative period | | 24-36 months postoperative period | |
| --- | --- | --- | --- | --- | --- | --- | --- | --- | --- | --- |
| Model | GLM sqrt-gamma | | GLM sqrt-gamma | | GLM log-normal | | GLM log-normal | | GLM log-normal | |
| Age (per 10 years, centered^a^) | 51.945  (4.976) | ** | 343.830  (25.106) | ** | 464.809  (95.839) | ** | 1230.299  (128.772) | ** | 965.468  (476.933) | * |
|  |  |  |  |  |  |  |  |  |  |  |
| **Outpatient versus inpatient setting** | **-107.454**  **(10.582)** | ****** | **-551.823**  **(55.575)** | ****** | **-439.133**  **(138.128)** | ****** | **-1096.622**  **(247.976)** | ****** | **-968.566**  **(430.334)** | ***** |
|  |  |  |  |  |  |  |  |  |  |  |
| Male versus female | -29.572 | ** | -36.364 |  | 214.429 |  | 294.466 |  | 216.027 |  |
|  | (10.782) |  | (55.697) |  | (147.476) |  | (206.517) |  | (426.472) |  |
|  |  |  |  |  |  |  |  |  |  |  |
| Urban versus rural | 37.415 | * | 141.838 |  | -166.318 |  | -334.454 |  | 131.024 |  |
|  | (15.328) |  | (78.161) |  | (177.554) |  | (328.557) |  | (530.849) |  |
|  |  |  |  |  |  |  |  |  |  |  |
| Material resources (versus least) | | | | | | | | | | |
| 2 | 6.385  (15.489) |  | 44.941  (91.050) |  | 48.803  (140.905) |  | -140.592  (323.300) |  | -794.822  (586.510) |  |
| 3 | 11.682  (16.593) |  | 68.945  (78.881) |  | 231.887  (148.128) |  | 535.916  (349.828) |  | -964.389  (775.404) |  |
| 4 | -9.997  (15.419) |  | 140.509  (89.454) |  | -26.559  (169.588) |  | -67.866  (357.529) |  | -719.439  (1014.399) |  |
| 5 (most) | 55.623  (22.416) | * | 238.982  (101.477) | * | 619.174  (204.806) | ** | 332.350  (433.704) |  | -1046.707  (904.279) |  |
|  |  |  |  |  |  |  |  |  |  |  |
| Racialized/newcomer (versus least) | | | | | | | | | | |
| 2 | 0.455 |  | -44.624 |  | 4.406 |  | 160.028 |  | 373.786 |  |
|  | (15.661) |  | (77.416) |  | (150.936) |  | (264.882) |  | (500.322) |  |
| 3 | 9.370 |  | -75.031 |  | 17.821 |  | -301.235 |  | 130.873 |  |
|  | (17.016) |  | (92.596) |  | (168.772) |  | (255.459) |  | (531.429) |  |
| 4 | 41.091 | * | 75.754 |  | 44.010 |  | 857.401 |  | 210.702 |  |
|  | (19.359) |  | (100.804) |  | (178.624) |  | (493.322) |  | (626.354) |  |
| 5 (most) | 92.984 | ** | 8.501 |  | 464.345 |  | 635.947 |  | 626.092 |  |
|  | (25.295) |  | (116.060) |  | (377.000) |  | (391.477) |  | (832.039) |  |
|  |  |  |  |  |  |  |  |  |  |  |
| Age and labor force (versus least) | | | | | | | | | | |
| 2 | 17.033 |  | -192.334 |  | 49.281 |  | -329.514 |  | 993.222 |  |
|  | (19.521) |  | (116.988) |  | (373.368) |  | (508.201) |  | (601.400) |  |
| 3 | 14.008 |  | -202.844 |  | -63.083 |  | -582.609 |  | 396.847 |  |
|  | (17.606) |  | (118.106) |  | (224.378) |  | (481.490) |  | (602.196) |  |
| 4 | 7.141 |  | -113.996 |  | 174.251 |  | -665.241 |  | 276.801 |  |
|  | (18.235) |  | (127.372) |  | (252.123) |  | (481.769) |  | (687.497) |  |
| 5 (most) | 30.447 |  | -118.663 |  | -26.267 |  | -146.736 |  | 959.489 |  |
|  | (19.130) |  | (135.652) |  | (219.354) |  | (478.662) |  | (701.039) |  |
|  |  |  |  |  |  |  |  |  |  |  |
| Households and dwellings (versus least) | | | | | | | | | | |
| 2 | -12.003 |  | 69.741 |  | -80.002 |  | 409.164 |  | 691.975 |  |
|  | (18.289) |  | (82.017) |  | (137.560) |  | (423.025) |  | (550.293) |  |
| 3 | -18.268 |  | 138.602 |  | 253.539 |  | 304.677 |  | 558.511 |  |
|  | (17.710) |  | (89.732) |  | (166.134) |  | (327.003) |  | (624.065) |  |
| 4 | -4.332 |  | 20.070 |  | 64.899 |  | 466.456 |  | 328.449 |  |
|  | (20.416) |  | (88.944) |  | (163.013) |  | (342.866) |  | (805.624) |  |
| 5 (most) | 27.847 |  | 441.020 | ** | 545.584 | ** | 844.131 | * | 377.707 |  |
|  | (20.886) |  | (155.623) |  | (211.698) |  | (364.263) |  | (608.246) |  |
|  |  |  |  |  |  |  |  |  |  |  |
| Total versus partial replacement | 45.112 |  | -2.204 |  | -449.354 |  | 152.879 |  | -1872.976 |  |
|  | (47.474) |  | (306.961) |  | (688.023) |  | (795.545) |  | (1508.977) |  |
|  |  |  |  |  |  |  |  |  |  |  |
| Comorbidity score (versus 0) | | | | | | | | | | |
| 1 | 256.015 | ** | 1578.865 | ** | 1433.297 | ** | 2859.699 | ** | 2618.716 | ** |
|  | (20.492) |  | (116.716) |  | (183.601) |  | (408.424) |  | (661.548) |  |
| 2 | 548.950 | ** | 3519.226 | ** | 2190.252 | ** | 5418.487 | ** | 7529.278 | ** |
|  | (90.617) |  | (587.914) |  | (441.321) |  | (1069.469) |  | (2812.914) |  |
| 3+ | 906.012 | ** | 5793.397 | ** | 7002.133 | * | 13814.832 | ** | 20826.289 |  |
|  | (177.249) |  | (1075.482) |  | (3046.847) |  | (4773.874) |  | (14243.351) |  |
|  |  |  |  |  |  |  |  |  |  |  |
| Fiscal year (versus 2019) |  |  |  |  |  |  |  |  |  |  |
| 2020 | 45.753 | * | 166.621 |  | 67.848 |  | -285.871 |  | -131.296 |  |
|  | (20.852) |  | (85.154) |  | (154.705) |  | (272.029) |  | (403.262) |  |
| 2021 | -8.068 |  | 492.726 | ** | 302.316 | * | 17.984 |  | - |  |
|  | (16.709) |  | (99.845) |  | (141.313) |  | (301.657) |  |  |  |
| 2022 | 10.643 |  | 500.987 | ** | - |  | - |  | - |  |
|  | (15.448) |  | (84.101) |  |  |  |  |  |  |  |
|  |  |  |  |  |  |  |  |  |  |  |
| Number of observations | 35835 |  | 35835 |  | 20450 |  | 14583 |  | 5433 |  |
| ** p<.01, * p<.05  ^a^ age was centered by subtracting the mean | | | | | | | | | | |

# Supplementary Table S3: Full regression marginal effects for knee arthroplasty

|  | Preoperative period | | 1-6 months postoperative period | | 6-12 months postoperative period | | 12-24 months postoperative period | | 24-36 months postoperative period | |
| --- | --- | --- | --- | --- | --- | --- | --- | --- | --- | --- |
| Model | GLM sqrt-gamma | | GLM log-gamma | | GLM sqrt-gamma | | GLM sqrt-gamma | | GLM log-gamma | |
| Age (per 10 years, centered^a^) | 64.273 | ** | 443.758 | ** | 374.887 | ** | 1003.088 | ** | 1424.531 | ** |
|  | (4.881) |  | (29.193) |  | (38.644) |  | (91.022) |  | (198.478) |  |
|  |  |  |  |  |  |  |  |  |  |  |
| **Outpatient versus inpatient setting** | **-91.610** | ****** | **-469.907** | ****** | **-408.879** | ****** | **-983.051** | ****** | **-1123.802** | ****** |
|  | **(8.652)** |  | **(47.359)** |  | **(74.356)** |  | **(180.613)** |  | **(350.205)** |  |
|  |  |  |  |  |  |  |  |  |  |  |
| Male versus female | -18.668 | * | 119.810 | * | 119.191 |  | -102.948 |  | -289.126 |  |
|  | (8.823) |  | (51.266) |  | (80.818) |  | (184.029) |  | (336.830) |  |
|  |  |  |  |  |  |  |  |  |  |  |
| Urban versus rural | 27.102 |  | -71.578 |  | 140.808 |  | 124.548 |  | 367.793 |  |
|  | (15.684) |  | (83.095) |  | (105.123) |  | (270.749) |  | (515.334) |  |
|  |  |  |  |  |  |  |  |  |  |  |
| Material resources (versus least) | | | | | | | | | | |
| 2 | -3.430 |  | 155.795 | * | -129.319 |  | 88.366 |  | 974.813 |  |
|  | (14.471) |  | (70.135) |  | (108.493) |  | (252.734) |  | (527.816) |  |
| 3 | -0.840 |  | 174.602 | * | 45.434 |  | 558.475 | * | 465.518 |  |
|  | (12.904) |  | (71.196) |  | (117.544) |  | (273.248) |  | (442.380) |  |
| 4 | -6.214 |  | 174.790 | * | -98.636 |  | 10.012 |  | 960.282 |  |
|  | (13.998) |  | (76.055) |  | (120.090) |  | (277.449) |  | (589.857) |  |
| 5 (most) | 6.569 |  | 364.159 | ** | 401.477 | * | 1177.344 | ** | 1624.460 | * |
|  | (15.428) |  | (88.542) |  | (183.972) |  | (369.292) |  | (669.035) |  |
|  |  |  |  |  |  |  |  |  |  |  |
| Racialized/newcomer (versus least) | | | | | | | | | | |
| 2 | 8.864 |  | 42.621 |  | 126.378 |  | 119.857 |  | 623.978 |  |
|  | (15.070) |  | (74.725) |  | (117.896) |  | (274.557) |  | (521.053) |  |
| 3 | -1.192 |  | 50.039 |  | 82.272 |  | -235.238 |  | -35.286 |  |
|  | (13.964) |  | (80.184) |  | (120.876) |  | (287.721) |  | (540.167) |  |
| 4 | 41.220 | ** | 200.055 | * | 433.929 | ** | 578.952 |  | 764.347 |  |
|  | (15.433) |  | (88.108) |  | (155.243) |  | (369.506) |  | (718.503) |  |
| 5 (most) | 57.604 | ** | 223.627 | * | 158.962 |  | 192.209 |  | 889.136 |  |
|  | (15.485) |  | (94.449) |  | (131.728) |  | (330.804) |  | (627.581) |  |
|  |  |  |  |  |  |  |  |  |  |  |
| Age and labor force (versus least) | | | | | | | | | | |
| 2 | 4.152 |  | -36.699 |  | 185.648 |  | 578.670 |  | 528.017 |  |
|  | (12.013) |  | (87.983) |  | (125.205) |  | (304.407) |  | (546.022) |  |
| 3 | 24.730 |  | -90.676 |  | 341.437 | ** | 380.126 |  | 948.009 |  |
|  | (15.219) |  | (86.751) |  | (127.942) |  | (299.632) |  | (638.177) |  |
| 4 | 13.873 |  | -44.533 |  | 169.807 |  | 181.705 |  | 64.982 |  |
|  | (13.546) |  | (90.714) |  | (122.471) |  | (289.450) |  | (572.908) |  |
| 5 (most) | 10.240 |  | -184.798 | * | 101.621 |  | 563.845 |  | 201.827 |  |
|  | (14.483) |  | (88.641) |  | (125.791) |  | (319.111) |  | (574.273) |  |
|  |  |  |  |  |  |  |  |  |  |  |
| Households and dwellings (versus least) | | | | | | | | | | |
| 2 | -7.069 |  | 104.952 |  | -7.673 |  | 2.405 |  | -267.675 |  |
|  | (14.897) |  | (75.162) |  | (106.429) |  | (297.318) |  | (484.975) |  |
| 3 | -2.496 |  | 196.837 | * | 171.112 |  | -31.830 |  | 669.034 |  |
|  | (14.251) |  | (76.727) |  | (123.318) |  | (309.170) |  | (612.420) |  |
| 4 | 19.344 |  | 224.868 | ** | 164.167 |  | 158.753 |  | 545.026 |  |
|  | (18.063) |  | (82.413) |  | (130.225) |  | (321.401) |  | (619.152) |  |
| 5 (most) | 51.089 | ** | 606.661 | ** | 588.496 | ** | 341.864 |  | 544.479 |  |
|  | (15.747) |  | (93.866) |  | (170.935) |  | (337.198) |  | (629.941) |  |
|  |  |  |  |  |  |  |  |  |  |  |
| Total versus partial replacement | -21.685 |  | -162.315 |  | -333.178 |  | -793.834 |  | -240.358 |  |
|  | (45.514) |  | (234.942) |  | (350.134) |  | (1031.820) |  | (1299.013) |  |
|  |  |  |  |  |  |  |  |  |  |  |
| Comorbidity score (versus 0) | | | | | | | | | | |
| 1 | 234.077 | ** | 1285.143 | ** | 1593.571 | ** | 2300.868 | ** | 3089.112 | ** |
|  | (20.887) |  | (82.204) |  | (200.529) |  | (277.315) |  | (670.063) |  |
| 2 | 424.499 | ** | 2452.967 | ** | 2718.973 | ** | 7287.638 | ** | 7039.485 | ** |
|  | (37.123) |  | (229.769) |  | (365.528) |  | (1435.069) |  | (2293.556) |  |
| 3+ | 635.172 | ** | 3993.127 | ** | 3377.012 | ** | 8545.147 | ** | 3424.177 |  |
|  | (79.342) |  | (542.249) |  | (952.512) |  | (2952.700) |  | (2641.020) |  |
|  |  |  |  |  |  |  |  |  |  |  |
| Fiscal year (versus 2019) |  |  |  |  |  |  |  |  |  |  |
| 2020 | 1.796 |  | 328.994 | ** | 210.582 |  | 57.051 |  | -113.570 |  |
|  | (26.520) |  | (125.370) |  | (121.284) |  | (267.475) |  | (348.749) |  |
| 2021 | -19.456 |  | 474.621 | ** | 362.382 | ** | 558.353 | * | - |  |
|  | (21.024) |  | (117.926) |  | (119.288) |  | (280.994) |  |  |  |
| 2022 | -34.168 |  | 378.204 | ** | - |  | - |  | - |  |
|  | (20.100) |  | (112.750) |  |  |  |  |  |  |  |
|  |  |  |  |  |  |  |  |  |  |  |
| Number of observations | 49597 |  | 49597 |  | 23836 |  | 15512 |  | 3977 |  |
| ** p<.01, * p<.05  ^a^ GLM log-normal  ^b^ OLS  ^c^ GLM square-root gamma  ^d^ age was centered by subtracting the mean | | | | | | | | | | |

# Supplementary Table S4: Costs in unmatched cohort by period and joint type

|  | **Hip arthroplasty** | |  | **Knee arthroplasty** | | |
| --- | --- | --- | --- | --- | --- | --- |
| **Time period** | **Inpatient (N=38593)** | **Outpatient (N=14905)** |  | **Inpatient (N=62707)** |  | **Outpatient (N=18877)** |
| **Preoperative (-30 to -2 days of surgery)** |  |  |  |  |  |  |
| N | 37544 | 14119 | 61310 | | 18255 | |
| Mean (SD) | $601 ($1390) | $392 ($945) | $595 ($1220) | | $432 ($1210) | |
| Median (Q1, Q3) | $333 [$182, $568] | $210 [$128, $378] | $354 [$195, $596] | | $251 [$145, $441] | |
| N missing (%) | 1049 (2.7%) | 786 (5.3%) | 1397 (2.2%) | | 622 (3.3%) | |
|  |  |  |  |  |  |  |
| **Perioperative (-1 to +30 days of surgery)** |  |  |  |  |  |  |
| N | 38593 | 14905 | 62707 | | 18877 | |
| Mean (SD) | $12400 ($8430) | $8000 ($5580) | $11700 ($7290) | | $7520 ($5490) | |
| Median (Q1, Q3) | $10900 [$9210, $13300] | $8150 [$3340, $10400] | $10400 [$8870, $12600] | | $7440 [$3190, $9750] | |
| N missing (%) | 0 (0%) | 0 (0%) | 0 (0%) | | 0 (0%) | |
|  |  |  |  |  |  |  |
| **1 to 6 months postoperative** |  |  |  |  |  |  |
| N | 38425 | 14789 | 62486 | | 18774 | |
| Mean (SD) | $3070 ($6770) | $1920 ($4970) | $3030 ($6300) | | $2240 ($4750) | |
| Median (Q1, Q3) | $1130 [$525, $2620] | $776 [$333, $1660] | $1260 [$615, $2790] | | $1020 [$492, $2130] | |
| N missing (%) | 168 (0.4%) | 116 (0.8%) | 221 (0.4%) | | 103 (0.5%) | |
|  |  |  |  |  |  |  |
| **6 to 12 months postoperative** |  |  |  |  |  |  |
| N | 31872 | 10160 | 52053 | | 12491 | |
| Mean (SD) | $2940 ($7140) | $1790 ($5350) | $2920 ($7560) | | $2110 ($5340) | |
| Median (Q1, Q3) | $833 [$262, $2300] | $444 [$146, $1310] | $915 [$308, $2430] | | $658 [$233, $1780] | |
| N missing (%) | 6721 (17.4%) | 4745 (31.8%) | 10654 (17.0%) | | 6386 (33.8%) | |
|  |  |  |  |  |  |  |
| **12 to 24 months postoperative** |  |  |  |  |  |  |
| N | 23712 | 5374 | 38690 | | 5543 | |
| Mean (SD) | $6000 ($13000) | $3430 ($8480) | $6340 ($13200) | | $4570 ($10800) | |
| Median (Q1, Q3) | $1850 [$607, $5230] | $1040 [$345, $2730] | $2210 [$783, $5850] | | $1520 [$565, $3850] | |
| N missing (%) | 14881 (38.6%) | 9531 (63.9%) | 24017 (38.3%) | | 13334 (70.6%) | |
|  |  |  |  |  |  |  |
| **24 to 36 months postoperative** |  |  |  |  |  |  |
| N | 14218 | 1640 | 24428 | | 1031 | |
| Mean (SD) | $6050 ($12700) | $3510 ($10400) | $6600 ($13400) | | $4380 ($10500) | |
| Median (Q1, Q3) | $1830 [$606, $5300] | $935 [$291, $2660] | $2300 [$842, $6070] | | $1540 [$471, $3690] | |
| N missing (%) | 24375 (63.2%) | 13265 (89.0%) | 38279 (61.0%) | | 17846 (94.5%) | |

# Supplementary Table S5: Sensitivity analysis for model

|  | Preoperative period | | Perioperative period | | 1-6 months postoperative period | | 6-12 months postoperative period | | 12-24 months postoperative period | | 24-36 months postoperative period | |
| --- | --- | --- | --- | --- | --- | --- | --- | --- | --- | --- | --- | --- |
| **Hip arthroplasty** |  | |  | |  | |  | |  | |  | |
|  | AME (SE) | p | AME (SE) | p | AME (SE) | p | AME (SE) | p | AME (SE) | p | AME (SE) | p |
| Matched cohort^a^ | -107.454 | ** | -3894.944 | ** | -551.823 | ** | -439.133 | ** | -1096.622 | ** | -968.566 | * |
|  | (10.582) |  | (76.444) |  | (55.575) |  | (138.128) |  | (247.976) |  | (430.334) |  |
|  |  |  |  |  |  |  |  |  |  |  |  |  |
|  | ATET (SE) | p | ATET (SE) | p | ATET (SE) | p | ATET (SE) | p | ATET (SE) | p | ATET (SE) | p |
| Sensitivity analysis (ATET)^b^ | -114.345 (10.353) | ** | -3982.543 (69.496) | ** | -595.753 (56.254) | ** | -524.697 (73.614) | ** | -1091.292 (140.768) | ** | -1147.357 (299.206) | ** |
|  |  |  |  |  |  |  |  |  |  |  |  |  |
| **Knee arthroplasty** |  | |  | |  | |  | |  | |  | |
|  | AME (SE) | p | AME (SE) | p | AME (SE) | p | AME (SE) | p | AME (SE) | p | AME (SE) | p |
| Matched cohort^a^ | -91.610 | ** | -3965.692 | ** | -469.907 | ** | -408.879 | ** | -983.051 | ** | -1123.802 | ** |
|  | (8.652) |  | (58.570) |  | (47.359) |  | (74.356) |  | (180.613) |  | (350.205) |  |
|  |  |  |  |  |  |  |  |  |  |  |  |  |
|  | ATET (SE) | p | ATET (SE) | p | ATET (SE) | p | ATET (SE) | p | ATET (SE) | p | ATET (SE) | p |
| Sensitivity analysis (ATET)^b^ | -161.450 (9.887) | ** | -4151.104 (49.337) | ** | -774.131 (42.502) | ** | -761.349 (67.097) | ** | -1769.327 (158.144) | ** | -2251.690 (336.003) | ** |
| ^a^ model is applied to the cohort truncated by propensity-score matching. Average marginal effects (AME) with the standard error (SE) are reported.  ^b^ model is applied to the cohort before matching. Inverse propensity-score weighting and regression adjustment are performed simultaneously and the SE is doubly-robust. The component of the model that produces the inverse propensity score was logistic regression. The component of the model that estimates the average treatment effect among the treated (ATET) was Poisson regression. The ATET was chosen since all outpatients could have received care in the inpatient setting, but not the reverse.  **p-value <0.0001 | | | | | | | | | | | | |
