# Supplementary material for: Cost savings of outpatient versus inpatient hip and knee arthroplasty in Ontario, Canada
Source: PLoS One. 2025 May 8;20(5):e0320255. doi: 10.1371/journal.pone.0320255 (PMC12061100; doi:10.1371/journal.pone.0320255)
Supplement: S1 Appendix — (DOCX) [file pone.0320255.s002.docx]

# Cost data sources

1. Hospital care. Inpatient (CIHI-DAD) and outpatient (CIHI-NACRS) records were linked to the Ontario Case Costing Initiative (OCCI) using a unique identifier (CIHI_key). For DAD, this was a 16-digit code produced by a concatenation of the healthcard province (5 if Ontario, Z otherwise), institution number (4 digits), fiscal year (4 digits), fiscal period (2 digits), batch number (2 digits), and abstract number (3 digits). For NACRS, this was an 18-digit code produced by a concatenation of the institution number (5 digits), fiscal year (4 digits), submission period (2 digits), and abstract number (7 digits). The OCCI includes direct and indirect costs and fixed and variable costs. Since the method relies on Management Information System (MIS) functional centre codes, the indirect costs include human resources (e.g., housekeeping), equipment (e.g., including depreciation), and other overhead (e.g., building and grounds expenses).^1^ However, some limitations must be acknowledged:
   1. Due to the COVID-19 pandemic, OCCI data for the 2020/21 and 2021/22 fiscal years were unavailable for use.
   2. The OCCI does not capture costs from all hospital corporations in the province but is believed to be representative with 52-53% of all hip/knee replacements being captured.^2^
   3. Using the 2019 and 2022 fiscal years only, we estimated that 70.1% of all DAD records and 53.3% of all NACRS records had a valid link to the OCCI.
   4. To address these limitations, we imputed the total costs for each DAD and NACRS record for the entire cohort. We imputed the cost using a frequency table distribution since we could not parameterize an accurate distribution to these cost data. We binned the costs at $1000 increments for inpatient (DAD) and $100 increments for outpatient visits (NACRS). A random number between 0 and 1 was assigned to each missing record, which was used to determine which cost bin that the record was assigned to (e.g., $5000-$5999 for DAD records; $400-$499 for NACRS records). To allow for some variability, another random number between 0 and 1 was generated to determine which specific value within that cost bin would be assigned (e.g. a random number of 0.82 corresponds to 82% of the distance between the lower and upper limits (e.g., $5820 or $482 in the examples above).
   5. To enable period-specific costs when an admission overlaps two different time periods, the total cost of the admission was converted into a daily cost from the admission date until the discharge date, inclusive. For example, if an admission record was associated with an admission date on postoperative day 180 and a discharge date on postoperative day 189 (10 days inclusive), then 40% (4 of 10 days) of the total cost of the admission would be assigned to the 1-6-month postoperative period (days 180-183) and 60% (days 184-189) to the 6-12-month postoperative period.
2. Physician billing costs were obtained from the Ontario Health Insurance Program (OHIP) database. These costs are remunerated to physicians for billable procedures covered under the OHIP. This includes procedures (e.g., surgeries), consultations or visits (e.g., general/family practitioner; specialists), and diagnostic imaging. Physician remuneration costs are negotiated between the Ontario Medical Association and the Ontario Ministry of Health. These are updated periodically and can be found in the Schedule of Benefits and fees (<https://www.ontario.ca/page/ohip-schedule-benefits-and-fees>).
3. Oral medication costs were captured from the ODB.
4. Rehabilitation care data were obtained from the National Rehabilitation Reporting System (NRS), which captures the length of stay of inpatient rehabilitation. The cost of care, however, was unavailable. Costs were instead estimated using the total length of stay for each patient, multiplied by the mean cost per day. The mean cost per day was extracted from the Healthcare Indicator Tool maintained by the Ontario Ministry of Health.^3^ Some limitations must be acknowledged:
   1. Inpatient rehabilitation costs did not distinguish between hip and knee replacement.
   2. Costs were provincial average (facility-specific or regional-specific costs were unavailable or incomplete).
   3. We used the Management Information Systems (MIS) functional centre code 7128120 for inpatient surgical rehabilitation by fiscal year: $497.06 (2019/20); $581.49 (2020/21); $648.64 (2021/22); $512.31 (2022/23); $516.29 (2023/24 Q2).
   4. Rehabilitation services provided in private clinics were unavailable.
   5. As with other hospitalization records, to enable period-specific costs, the total cost of admission was converted into a daily cost from the admission date until the discharge date, inclusive.
5. Home care cost data were unavailable directly, but the number of units (visits or hours) by each health service provider type (e.g. nurse, physiotherapist, occupational therapist, nutritionist/dietitian) was available. To translate this into costs, the number of visits or hours were multiplied by their unit costs. Unit costs were derived from the Ontario Healthcare Financial and Statistical System. Costs were available for the 2019/20 fiscal year. Facility-level or geographic variation in unit costs was unavailable or incomplete, so the provincial average cost was used instead.

# References

1. Canadian Patient Cost Database Technical Document: MIS Patient Costing Methodology, January 2019. 2019.

2. Quality-Based Procedures: Clinical Handbook for Primary Hip and Knee Replacement. *Health Quality Ontario; Ministry of Health and Long-Term Care*. November 2013. http://www.hqontario.ca/evidence/publications-and-ohtac-recommendations/clinical-handbooks. Accessed May 21, 2024.

3. Health Data Branch Web Portal | Ministry of Health. https://hsim.health.gov.on.ca/hdbportal/. Accessed July 25, 2024.
