# Supplementary material for: Cost savings of outpatient versus inpatient hip and knee arthroplasty in Ontario, Canada
Source: PLoS One. 2025 May 8;20(5):e0320255. doi: 10.1371/journal.pone.0320255 (PMC12061100; doi:10.1371/journal.pone.0320255)
Supplement: S1 Checklist — (DOCX) [file pone.0320255.s003.docx]

STROBE Statement—checklist of items that should be included in reports of observational studies

|  | Item No. | Recommendation | Page  No. | Relevant text from manuscript |
| --- | --- | --- | --- | --- |
| **Title and abstract** | 1 | (*a*) Indicate the study’s design with a commonly used term in the title or the abstract | 2 | This was a population-based retrospective cohort study. |
|  |  | (*b*) Provide in the abstract an informative and balanced summary of what was done and what was found | 2 | Methods and results section of abstract |
| Introduction | | | |  |
| Background/rationale | 2 | Explain the scientific background and rationale for the investigation being reported | 3-4 | Since the COVID-19 pandemic, there has been a rise in the number of outpatient hip/knee replacements performed. However, there exists a gap in the literature on the total cost-savings and quality differences between inpatient and outpatient hip and knee replacement |
| Objectives | 3 | State specific objectives, including any prespecified hypotheses | 4 | Purpose: to estimate the cost savings associated with outpatient versus inpatient hip and knee arthroplasty across both perioperative and post-perioperative timeframes, using population-based data from Ontario. |
| Methods | | | |  |
| Study design | 4 | Present key elements of study design early in the paper | 4-5 |  |
| Setting | 5 | Describe the setting, locations, and relevant dates, including periods of recruitment, exposure, follow-up, and data collection | 4 | Inpatient and outpatient arthroplasties performed in Ontario hospitals between April 1, 2019 and March 31, 2022.  Data were extracted in March 2024. |
| Participants | 6 | (*a*) *Cohort study*—Give the eligibility criteria, and the sources and methods of selection of participants. Describe methods of follow-up  *Case-control study*—Give the eligibility criteria, and the sources and methods of case ascertainment and control selection. Give the rationale for the choice of cases and controls  *Cross-sectional study*—Give the eligibility criteria, and the sources and methods of selection of participants | 4-5 | Patient inclusion and exclusion criteria. Also Fig 1. |
|  |  | (*b*) *Cohort study*—For matched studies, give matching criteria and number of exposed and unexposed  *Case-control study*—For matched studies, give matching criteria and the number of controls per case | 9-10 | Information on matching described here. Results of balance in Supplementary Tables S1 and S2 |
| Variables | 7 | Clearly define all outcomes, exposures, predictors, potential confounders, and effect modifiers. Give diagnostic criteria, if applicable | 5-9 | Covariates identified and measured (pages 5-6).  Outcome identified and measure din detail (pages 6-9) |
| Data sources/ measurement | 8* | For each variable of interest, give sources of data and details of methods of assessment (measurement). Describe comparability of assessment methods if there is more than one group | 5-9 |  |
| Bias | 9 | Describe any efforts to address potential sources of bias | n/a |  |
| Study size | 10 | Explain how the study size was arrived at | Fig 1 |  |

Continued on next page

| Quantitative variables | 11 | Explain how quantitative variables were handled in the analyses. If applicable, describe which groupings were chosen and why |  |  |
| --- | --- | --- | --- | --- |
| Statistical methods | 12 | (*a*) Describe all statistical methods, including those used to control for confounding | 9-11 | Statistical methods subsection |
|  |  | (*b*) Describe any methods used to examine subgroups and interactions | n/a |  |
|  |  | (*c*) Explain how missing data were addressed | Fig 1 | Analysis was performed on complete-case. |
|  |  | (*d*) *Cohort study*—If applicable, explain how loss to follow-up was addressed  *Case-control study*—If applicable, explain how matching of cases and controls was addressed  *Cross-sectional study*—If applicable, describe analytical methods taking account of sampling strategy | 9-10 | Matching is described |
|  |  | (*e*) Describe any sensitivity analyses | 11 | In sensitivity analysis, we used a model that is less sensitive to mis-specification (the IPW-RA model) |
| Results | | | | |
| Participants | 13* | (a) Report numbers of individuals at each stage of study—eg numbers potentially eligible, examined for eligibility, confirmed eligible, included in the study, completing follow-up, and analysed | Fig 1 |  |
|  |  | (b) Give reasons for non-participation at each stage | Fig 1 |  |
|  |  | (c) Consider use of a flow diagram | Fig 1 |  |
| Descriptive data | 14* | (a) Give characteristics of study participants (eg demographic, clinical, social) and information on exposures and potential confounders | Table S1-S2 |  |
|  |  | (b) Indicate number of participants with missing data for each variable of interest | Fig 1 | Complete case |
|  |  | (c) *Cohort study*—Summarise follow-up time (eg, average and total amount) | n/a |  |
| Outcome data | 15* | *Cohort study*—Report numbers of outcome events or summary measures over time | n/a | Temporal component n/a |
|  |  | *Case-control study—*Report numbers in each exposure category, or summary measures of exposure | n/a |  |
|  |  | *Cross-sectional study—*Report numbers of outcome events or summary measures | n/a |  |
| Main results | 16 | (*a*) Give unadjusted estimates and, if applicable, confounder-adjusted estimates and their precision (eg, 95% confidence interval). Make clear which confounders were adjusted for and why they were included | Table 1-2  Pages 12-15 |  |
|  |  | (*b*) Report category boundaries when continuous variables were categorized | n/a |  |
|  |  | (*c*) If relevant, consider translating estimates of relative risk into absolute risk for a meaningful time period | Table 1-2  Pages 12-15 | Average marginal effects |

| Other analyses | 17 | Report other analyses done—eg analyses of subgroups and interactions, and sensitivity analyses | 16 | Sensitivity analysis on statistical model |
| --- | --- | --- | --- | --- |
| Discussion | | | | |
| Key results | 18 | Summarise key results with reference to study objectives | 16 |  |
| Limitations | 19 | Discuss limitations of the study, taking into account sources of potential bias or imprecision. Discuss both direction and magnitude of any potential bias | 19 | Limitations subsection of discussion |
| Interpretation | 20 | Give a cautious overall interpretation of results considering objectives, limitations, multiplicity of analyses, results from similar studies, and other relevant evidence | 20 | Conclusion subsection of discussion |
| Generalisability | 21 | Discuss the generalisability (external validity) of the study results | 19 | However, in appropriately selected patients, outpatient arthroplasty is cost-saving from the perspective of the healthcare system and we expect these findings to generalize to jurisdictions having a similar healthcare system as Ontario. |
| Other information | |  | | |
| Funding | 22 | Give the source of funding and the role of the funders for the present study and, if applicable, for the original study on which the present article is based | 1 | Funding: No specific sources of funding were used |

*Give information separately for cases and controls in case-control studies and, if applicable, for exposed and unexposed groups in cohort and cross-sectional studies.

**Note:** An Explanation and Elaboration article discusses each checklist item and gives methodological background and published examples of transparent reporting. The STROBE checklist is best used in conjunction with this article (freely available on the Web sites of PLoS Medicine at http://www.plosmedicine.org/, Annals of Internal Medicine at http://www.annals.org/, and Epidemiology at http://www.epidem.com/). Information on the STROBE Initiative is available at www.strobe-statement.org.
